# Characterization of the interferences of systemic azole antifungal drugs with adrenal steroid biosynthesis using H295R cells and enzyme activity assays

**DOI:** 10.1016/j.crtox.2023.100119

**Published:** 2023-08-14

**Authors:** Marie-Christin Jäger, Friedrich L. Joos, Denise V. Winter, Alex Odermatt

**Affiliations:** Swiss Centre for Applied Human Toxicology and Division of Molecular and Systems Toxicology, Department of Pharmaceutical Sciences, University of Basel, Klingelbergstrasse 50, 4056 Basel, Switzerland

**Keywords:** H295R, Azole antifungals, Hypertension, Hypokalemia, Posaconazole, Itraconazole, Steroidogenesis

## Abstract

Azole antifungals, designed to inhibit fungal CYP51, have a liability to inhibit human CYP enzymes. Whilst drug-metabolizing CYPs are covered in preclinical safety assessment, those metabolizing endogenous bioactive molecules are usually not. Posaconazole and itraconazole were recently found to cause pseudohyperaldosteronism with hypokalemia and hypertension by inhibiting CYP11B1-dependent adrenal cortisol biosynthesis. Because this was overlooked in preclinical safety assessment, the present study tested whether applying adrenal carcinoma H295R cells could have predicted this liability and whether other systemic triazole antifungals interfere with adrenal steroidogenesis. Forskolin-stimulated H295R cells were exposed to systemic triazole antifungals that are currently used, and key adrenal steroids were quantified by UHPLC-MS/MS. To support the findings from the H295R model, activity assays for steroidogenic enzymes were performed. The analysis of the steroid profiles and product/substrate ratios predicted the CYP11B1 and CYP11B2 inhibition by posaconazole and itraconazole. Comparison of their steroid profiles allowed distinguishing their effects and suggested inhibition of adrenal androgen synthesis by posaconazole but not itraconazole, which was confirmed by CYP17A1 17,20-lyase activity measurements. In line with clinical observations, there was no evidence from these experiments for an inhibition of either CYP11B1/2 or CYP17A1 by voriconazole, fluconazole or isavuconazole. However, itraconazole and isavuconazole exerted an overall inhibition of steroidogenesis by a mechanism warranting further investigations. In conclusion, analyses of steroid profiles from the H295R assay and product/substrate ratios provide important information on the interference of a chemical with adrenal steroidogenesis and the underlying mechanism. This approach facilitates prioritization of further investigations, including enzyme expression and activity studies.

## Highlights

• Pseudohyperaldosteronism by azole antifungals can be predicted using H295R cells.
• The H295R assay distinguishes inhibitory effects of posaconazole and itraconazole.
• Inhibition of CYP17A1 17,20-lyase by posaconazole may contribute to gynecomastia.
• Isavuconazole and itraconazole concentrations  ≥ 1 µM block overall steroidogenesis.

## Graphical abstract

**Figure:**
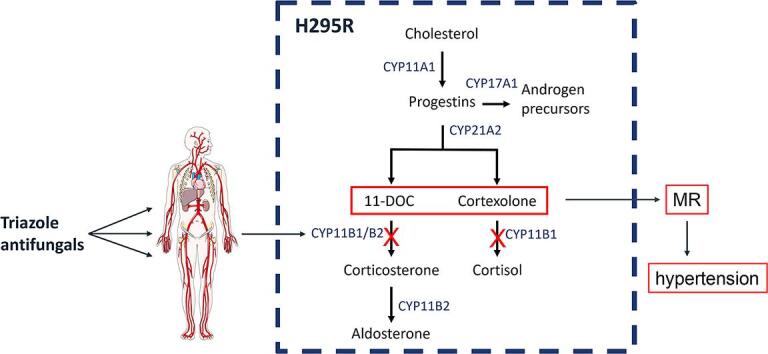

## Introduction

**Fig. 1:**
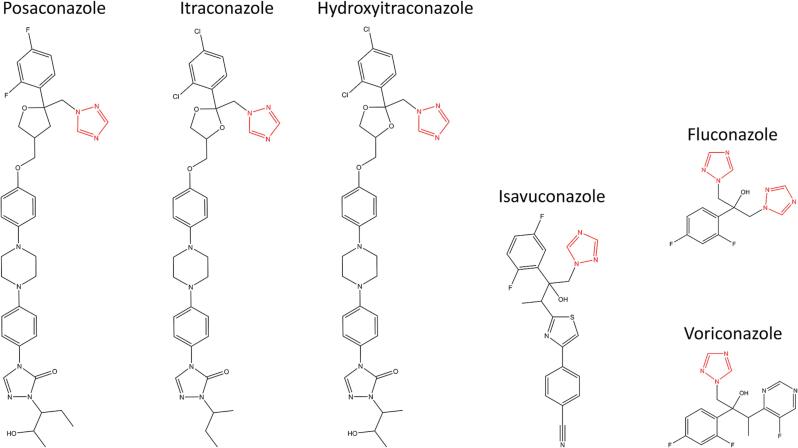
Structures of the systemic triazole antifungals with the common triazole moiety marked in red. The major itraconazole metabolite hydroxyitraconazole is also indicated as it retains bioactivity and was included in the enzyme activity assays.

Azole antifungals are applied as first-line therapy for fungal infections. They inhibit the fungal cytochrome P450 (CYP) enzyme 14α-lanosterol demethylase (CYP51) and prevent the formation of ergosterol, thereby disrupting the fungal cell membrane integrity and resulting in the accumulation of toxic sterol intermediates (Carrillo-Muñoz et al., 2006, Hof, 2006, Campoy and Adrio, 2017). To date, three classes of azole antifungals have been approved for clinical application: imidazoles (which are predominantly used as topical agents), triazoles, and the most recently introduced tetrazole oteseconazole (also known as VT-1161) (Allen et al., 2015, Hoy, 2022). Systemic imidazole antifungals had a high failure rate and showed unacceptable side-effects including cardiac toxicity and neurologic impairments (Allen et al., 2015). The most prominent imidazole antifungal initially used systemically is ketoconazole. During clinical administration, patients suffered from severe side-effects, including pseudohyperaldosteronism with hypokalemia and hypertension as well as gynecomastia and decreased libido (DeFelice et al., 1981, Pont et al., 1982, Schürmeyer and Nieschlag, 1982, Grosso et al., 1983, Pont et al., 1984, Aabo and De Coster, 1987, Leal-Cerro et al., 1989, Engelhardt et al., 1991, Gupta et al., 2015). Later, triazole antifungals were developed with improved safety and pharmacokinetic profiles compared to imidazoles, while maintaining or enhancing the antifungal spectrum. This class includes voriconazole, fluconazole, itraconazole, posaconazole and isavuconazole (for structures, see Fig. 1) (Allen et al., 2015, Campoy and Adrio, 2017). Despite the well-documented severe side-effects due to the disruption of adrenocortical functions by ketoconazole, the newly developed azole antifungals were not evaluated for their potential to disrupt adrenal steroidogenesis prior to market approval.

Several case reports emphasized the risk of posaconazole and itraconazole for pseudohyperaldosteronism with typically low renin and aldosterone levels and hypokalemia and hypertension (Antonarakis et al., 2013, Denolle et al., 2014, Thompson et al., 2017, Barton et al., 2018, Boughton et al., 2018, Hoffmann et al., 2018, Wassermann et al., 2018, Lee et al., 2019, Agarwal et al., 2020, Parker et al., 2020, Teaford et al., 2020, Brandi et al., 2021, Marpole et al., 2021, Villar-Prados et al., 2021). *In vitro* studies elucidated the mechanism of action, demonstrating inhibition of 11β-hydroxylases (CYP11B1 and CYP11B2) and 11β-hydroxysteroid dehydrogenase type 2 (11β-HSD2) as the primary cause for posaconazole- and itraconazole-induced pseudohyperaldosteronism (Beck et al., 2017, Beck et al., 2020a). Interestingly, itraconazole was found to exert more potent inhibition towards 11β-HSD2 but weaker inhibition of CYP11B1 compared with posaconazole.

**Fig. 2:**
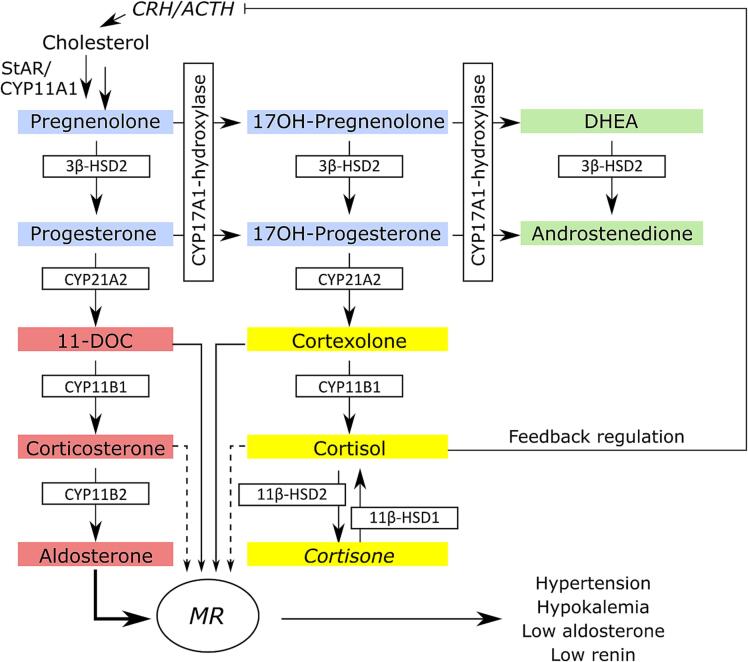
Schematic representation of adrenal steroidogenesis. The formation of progestins (blue), mineralocorticoids (red), glucocorticoids (yellow) and adrenal androgens (green) and the involved enzymes is shown. Steroidogenesis underlies a negative feedback regulation, involving a downregulation of adrenocorticotrophic hormone (ACTH) and corticotrophin releasing hormone (CRH) in response to high cortisol levels. Aldosterone is the most potent agonist of the mineralocorticoid receptor (MR), whilst 11-deoxycorticosterone (11-DOC) and cortexolone are moderate agonists. Corticosterone and cortisol are potent activators in situations of inhibited 11β-HSD2. The peripheral interconversion of cortisol and cortisone by 11β-HSDs is indicated. Cortisone, shown in *italic*, is not produced by the adrenals. Following 11β-HSD2 or CYP11B1 inhibition, an excessive MR activation leads to hypokalemia, hypernatremia and water retention, with low aldosterone and low renin hypertension.

11β-HSD2 catalyzes the conversion of potent 11β-hydroxyglucocorticoids (cortisol, corticosterone) to their inactive 11-oxo metabolites (cortisone, 11-dehydrocorticosterone) in peripheral tissues such as kidney and colon. Even though aldosterone and the about 1000 times more abundant cortisol have similar affinities for the mineralocorticoid receptor (MR), aldosterone can access this receptor and regulate electrolyte concentrations due to the local inactivation of 11β-hydroxyglucocorticoids by 11β-HSD2 (Arriza et al., 1987, Edwards et al., 1988, Funder et al., 1988). 11β-HSD2 inhibition results in glucocorticoid-induced MR activation, with low renin and low aldosterone, ultimately causing sodium and water retention, hypokalemia and hypertension. CYP11B1 and CYP11B2 essentially catalyze the last step of the biosynthesis of cortisol (and corticosterone) and aldosterone, respectively (Fig. 2) (Miller and Auchus, 2011). Unspecific CYP11B inhibition results in an ablation of aldosterone production. Furthermore, due to the feedback activation of adrenal steroidogenesis attempting to maintain normal circulating cortisol levels, the concentrations of the moderate MR agonists cortexolone (known as 11-deoxycortisol) and 11-deoxycorticosterone (11-DOC) can reach supra-physiological levels (Zipser et al., 1980, Hellal-Levy et al., 1999, Miller and Auchus, 2011). The excessive levels of CYP11B substrates, mainly of 11-DOC, then result in pseudohyperaldosteronism, as seen after 11β-HSD2 inhibition (Tsigos and Chrousos, 2002). A similar phenotype can be caused by inhibition of CYP17A1 17α-hydroxylase (Miller and Auchus, 2011, Beck et al., 2020b), resulting in a feedback activation of steroidogenesis and enhanced mineralocorticoid levels.

Patients suffering from pseudohyperaldosteronism following treatment with posaconazole or itraconazole exhibited elevated serum cortexolone and 11-DOC, indicating CYP11B1/2 inhibition (Thompson et al., 2017, Barton et al., 2018, Boughton et al., 2018, Agarwal et al., 2020). In some patients cortexolone and 11-DOC levels were not altered, and inhibition of 11β-HSD2 was proposed to be responsible for the observed pseudohyperaldosteronism (Thompson et al., 2017, Thompson et al., 2019, Parker et al., 2020). The cortisol to cortisone ratio was determined in some case studies to assess 11β-HSD2 activity, indicating its inhibition in>70% of the documented cases (Thompson et al., 2017, Boughton et al., 2018, Wassermann et al., 2018, Thompson et al., 2019, Agarwal et al., 2020, Thompson et al., 2020). To date, there are neither case reports providing *in vivo* evidence for an inhibition of CYP17A1 17α-hydroxylase by the systemic triazole antifungals nor cases indicating disturbances of adrenal steroidogenesis by voriconazole, fluconazole and isavuconazole.

The interference of azole antifungal drugs with adrenal steroidogenesis turned out to be an underestimated risk. Currently, pre-clinical drug safety assessment does not necessarily cover adrenal steroidogenesis. The Food and Drug Administration (FDA) adjusted their guidance for industry, suggesting to test some CYP enzymes “involved in anabolism and catabolism of steroid hormones” (FDA, 2015). However, there are currently no procedures or guidelines that define testing workflows for steroidogenic CYPs or for non-CYP enzymes involved in steroid metabolism. The present project applied the adrenal carcinoma H295R cell model to: 1) test whether the risk of pseudohyperaldosteronism by posaconazole and itraconazole *via* CYP11B1 inhibition could have been predicted, 2) analyze whether posaconazole and itraconazole exert additional effects on the adrenal steroid profile and whether their pattern can be distinguished, and 3) assess whether the triazoles voriconazole, fluconazole and isavuconazole exert yet unknown effects on the adrenal steroidogenesis. The H295R steroidogenesis cell model was initially introduced by the Organization of Economic Cooperation and Development (OECD) and the USEPA Endocrine Disruptor Program (EDSP) in the OECD test guideline 456 to detect chemicals causing changes in testosterone and estradiol levels (Hecker et al., 2011, OECD, 2011). In the present study, a modified protocol using forskolin-stimulated cells to facilitate the detection of inhibitors and using LC-MS/MS to quantify mineralocorticoids, glucocorticoids, progestins and adrenal androgens as described earlier (Strajhar et al., 2017, Patt et al., 2020) was applied. This approach was complemented using cell-free enzyme activity assays based on cells over-expressing recombinant enzymes of interest to validate hypotheses on enzyme inhibition drawn from the H295R cell experiments.

## Material and methods

### Chemicals and solvents

Posaconazole (CAS: 171228–49-2), fluconazole (CAS: 86386–73-4), voriconazole (CAS: 137234–62-9), isavuconazole (CAS: 241479–67-4), prochloraz (CAS: 67747–09-5), ketoconazole (CAS: 65277–42-1), itraconazole (CAS: 84625–61-6), bisphenol A (CAS: 80–05-7), 11-DOC (CAS: 64–85-7), [2,2,4,6,6,21,21,21-D8]-17α-hydroxyprogesterone (CAS: 850023–80-2), [2,2,3,4,4,6-D6]-dehydroepiandrosterone (CAS: 1261254–39-0), [2,2,4,4,6,6,21,21-D7]-aldosterone (CAS: 1261254–31-2), [2,2,4,6,6-D5]-cortexolone, MOPS (CAS: 1132–61-2), dithiothreitol (DTT, CAS: 3483–12-3), and Tris-Base (CAS: 77–86-1) were obtained from Sigma-Aldrich (Buchs, Switzerland). Hydroxyitraconazole (OH-itraconazole, CAS: 112559–91-8) was purchased from Carbosynth (Staad, Switzerland), [1,2,6,7-3H(N)]-progesterone (CAS: 94391–12-5) was purchased from Perkin Elmer (Schwerzenbach, Switzerland). 17α-hydroxypregnenolone (CAS: 387–79-1) and 20*S*-hydroxy-cholesterol (20OH-cholesterol, CAS:516–72-3) were obtained from MedChemExpress (Monmouth Junction, USA). Androstenedione (CAS: 63–05-8), dehydroepiandrosterone (CAS: 53–43-0), corticosterone (CAS: 50–22-6), cortexolone (CAS:152–58-9), aldosterone (CAS: 52–39-1), cortisol (CAS: 50–23-7), progesterone (CAS: 57–83-0) and 17α-hydroxyprogesterone (CAS: 68–96-2) were purchased from Steraloids (Newport, RI, USA) and [2,2,6,6,17,21,21,21-D8]-progesterone (unlabeled CAS 57–83-0) was purchased from Cambridge isotope laboratories (Andover, MA, USA). Potassium chloride (CAS: 7447–40-7), EGTA (CAS: 67–42-5), and EDTA (60–00-4) were ordered from Merck (Darmstadt, Germany), magnesium chloride (CAS: 7791–18-6) and sucrose (CAS: 57–50-1) from Fluka (North Carolina, USA), and HEPES buffer (1 M, CC grade) from BioConcept (Allschwil, Switzerland). All plasmids used to transfect cells were obtained from GeneScript (Piscataway, USA). Stock solutions of test substances and steroids were prepared in methanol (Biosolve, Dieuze, France) or dimethyl sulfoxide (DMSO, CAS: 67–68-5, Acros Organics, Geel, Belgium). Ethyl acetate and acetonitrile were ordered from Fisher Scientific (Reinach, Switzerland) and Scharlau (Barcelona, Spain), respectively.

### Cell culture

H295R human adrenocarcinoma, V79-4 and COS-1 cells were purchased from American Type Culture Collection (ATCC, Manassas, VA, USA). H295R cells were cultured in Dulbecco‘s modified Eagle’s medium (DMEM)/Ham‘s nutrient mixture F-12 (1:1, v/v) (Live Technologies, Zug, Switzerland) containing 1% (v/v) ITS + Premix (BD Bioscience, Bedford, MA, USA), 2.5% (v/v) Nu-serum (Lot: 0154001, BD Bioscience), 100 U/mL penicillin/100 µg/mL streptomycin (Sigma-Aldrich) and HEPES buffer, pH 7.4.

V79-4 and COS-1 cells were cultured in DMEM high glucose (Sigma-Aldrich) supplemented with 4 mM L-glutamine, 100 U/mL penicillin/100 µg/mL, 10% (v/v) fetal bovine serum (FBS, Biowest Nuaillé, France) and 1 mM sodium pyruvate (Sigma-Aldrich).

### Steroidogenesis assay in H295R cells

The steroidogenesis assay was performed as recommended by the OECD test guideline 456 (OECD, 2011) with modifications described earlier (Patt et al., 2020). Briefly, 200,000 cells/mL between passages 5 and 10 were seeded in a 24-well plate. After 24 h, the medium was replaced by fresh medium containing reference or test compounds. In contrast to the protocol described in the OECD guideline where the cells are used in the basal state, steroidogenesis was stimulated with 10 μM of the adenylyl cyclase inducer forskolin (Pinto et al., 2008), in order to facilitate the detection of inhibitors. As a time zero control, complete medium was incubated without cells. After 48 h of incubation, the cell culture supernatant was collected and stored at −20 °C until steroid quantification was performed by ultra-high liquid chromatography tandem mass spectrometry (UHPLC-MS/MS). All experiments were performed three times independently in technical duplicates.

### Cell viability assay in H295R cells

Cell viability was determined in H295R cells using the 2,3-bis(2-methoxy-4-nitro-5-sulfophenyl)–2H-tetrazolium-5-carboxanilide (XTT) assay. Cells (30,000 per well) were seeded in a 96-well plate. After 24 h, the medium was replaced by medium containing the test compounds in concentrations ranging from 0.1 μM to 10 μM in the presence of 10 μM forskolin. After 48 h, 25 µL of a XTT/phenazine methosulfate solution (Sigma-Aldrich) (1 mg/mL and 7.5 μg/mL, respectively) were added to each well, followed by 2 h of incubation. Absorbance measurements were performed at 450 nm and 650 nm. The experiments were performed three times independently in triplicates.

### Targeted steroid quantification

Steroid hormones were measured using UHPLC-MS/MS as described earlier (Strajhar et al., 2017, Akram et al., 2019), with minor adaptions. H295R cell culture supernatant, 450 µL, was spiked with aldosterone-D7, cortexolone-D5 (also called 11-deoxycortisol-D5), corticosterone-D8, DHEA-D6, 17-hydroxyprogesterone-D8 and progesterone-D8 to final concentrations of 0.25 ng/mL each, except for DHEA-D6 which had a final concentration of 1 ng/mL. Using water, the sample volume was adapted to 1 mL. Samples were extracted using Oasis HLB 3 cc SPE cartridge (30 mg, 30 μm particle size, Waters, Massachusetts, USA). Columns were preconditioned with 1 mL ethyl acetate and water prior to application of samples. After sample loading, columns were washed three times with 1 mL methanol (10% v/v) and water before samples were eluted applying 0.75 mL ethyl acetate twice. Eluates were evaporated to dryness and reconstituted in 50 µL methanol. Samples were applied to a reverse phase column (Waters Acquity UPLC BEH C18, 1.7 μm, 2.1 mm × 150 mm). Steroids were separated by an Agilent 1290 UHPLC system and detected using an Agilent 6490 triple quadrupole mass spectrometer with a jet-stream electrospray ionization source. Applied mobile phases consisted of water, acetonitrile and formic acid (95/5/0.1; v/v/v and 5/95/0.1; v/v/v). For data analysis, Hunter software version B.09.00 (Agilent Technologies) was used. Lower limit of detection (LOD), lower limit of quantification (LLOQ), and upper limit of quantification (ULOQ) are shown in Table 1.

**Table 1:** Sensitivity of the UHPLC-MS/MS method for the steroids quantified.

concentrations in ng/mL	progesterone	17α-hydroxy-progesterone	11-DOC	corticosterone	aldosterone	cortexolone	cortisol	DHEA	androsten-dione
LOD	0.019	0.195	0.526	0.036	0.031	1.061	0.559	0.301	0.281
LLOQ	0.055	0.341	1.082	0.194	0.064	2.047	0.988	0.814	0.601
ULOQ	4	25	100	50	3	200	100	25	50

### CYP11A1 assay based on mitochondrial fraction of V79-4 cells

Mitochondria were prepared as described previously with modifications (Frezza et al., 2007). Briefly, V79-4 cells (3,000,000) were seeded 4 h prior to transfection with 4 µg of expression plasmid for CYP11A1 and adrenodoxin (GeneScript Piscataway, USA, clone IDs OHu18602D and OHu55929D) using polyethylemine (PEI) in a PEI/DNA ratio of 3:1 (w/w). At 4 h post-transfection, medium was changed and cells were incubated overnight. Cells were collected in ice cold phosphate buffered saline (PBS) and centrifuged at 600 × g for 10 min at 4 °C. The pellet was resuspended in ice cold isolation buffer (10 mM Tris/MOPS, pH 7.4, 1 mM EGTA/Tris, pH 7.4, 200 mM sucrose in water) and transferred to a glass potter, followed by 35 S using a 2 cm^3^ glass Teflon pestle. The homogenate was centrifuged for 10 min at 600 × g and 4 °C. The supernatant was collected and centrifuged for 10 min at 7000 × g at 4 °C. The pellet was washed and resuspended in 200 μL ice cold isolation buffer. After centrifugation, the pellet containing the mitochondrial fraction was resuspended in 30 μL reaction buffer (25 mM HEPES pH 7.4, 0.1 mM DTT, 0.1 mM EDTA, 4 mM MgCl_2_) and the protein content was determined by the BCA assay according to the manufacturer (Pierce BCA protein assay kit, Thermo Scientific, Rockford, USA). Bovine serum albumin provided with the kit was used as reference protein and absorbance was measured using a BioTek Cytation5 Imaging Reader (Agilent BioTek).

For the CYP11A1 activity assay, 500 µg/mL of freshly prepared mitochondrial fraction were pre-incubated on ice with reaction buffer, 1 mM NADPH and either vehicle (0.1% DMSO), 10 µM of the positive control ketoconazole (Mast et al., 2013) or the triazole antifungals for 10 min. The reaction was started by adding the substrate 20*S*-hydroxycholesterol at a final concentration of 2 µM in a reaction volume of 50 µL. After 4 h of incubation at 37 °C, the reaction was stopped by adding 50 µL methanol and shock-freezing in liquid nitrogen. Samples were stored at −80 °C until sample preparation for pregnenolone detection using an ELISA kit (Alpco, Salem, USA). Sample preparation included evaporation of samples to dryness, reconstitution in 6 µL methanol and 1:25 (v:v) dilution in water. The ELISA was performed according to the manufacturer and absorbance was measured using a BioTek Citation5 imaging reader.

### Preparation of microsomes from transiently transfected COS-1 cells

Microsomes were prepared from COS-1 cells transiently transfected with plasmids for either CYP17A1 and POR for CYP17A1-hydroxylase assay (human CYP17A1 was available in our own plasmid collection after cloning into pcDNA3, POR was ordered from Genescript, Piscataway, USA, clone ID OHu25584C), CYP17A1, POR and cytochrome b5 for CYP17A1 17,20-lyase assay (cytochrome b5 was from Genescript, clone ID OHu09495D), POR and CYP21A2 (Genescript, CYP21A2 clone ID OHu18602D), or 3β-HSD2 (Genescript, clone ID OHu14034D). Transfection was performed using PEI and equal amounts of required plasmids with a total DNA amount of 8 µg per transfection of 3,000,000 cells. After overnight incubation, cells were washed with prewarmed PBS before adding ice-cold PBS. Cells were collected and centrifuged at 600 × g for 4 min at 4 °C, washed with ice cold PBS and centrifuged again. The pellet was resuspended in a resuspension buffer (20 mM Tris, pH 7.5, 50 mM KCl, 2 mM MgCl_2_, 0.25 M sucrose). Cells were disrupted with 30 S by a glass potter (2 cm^3^) as described above. The homogenate was centrifuged for 20 min at 12,000 × g and 4 °C. Microsomes were pelleted by ultracentrifugation at 100,000 × g for 1 h at 4 °C. The pellet was resuspended in reaction buffer and the protein concentration determined using the Pierce Protein BCA kit as described above.

### CYP17A1 17α-hydroxylase and CYP21A2 activity assay

Microsomal protein (250 µg/mL) was preincubated with 1 mM NADPH and the compound of interest in reaction buffer for 10 min. The reaction was started by adding progesterone spiked with a radioactive tracer (final activity 0.025 mCi) to a final concentration of 500 nM. After 10 min of incubation at 37 °C the reaction was stopped by adding enzyme substrate and product at a final concentration of 1 mM in methanol, followed by shock-freezing in liquid nitrogen. Steroids were separated by thin layer chromatography on SIL G-25 UV254 TLC plates (Macherey-Nagel, Düren, Germany) using chloroform and ethyl acetate (1:4; v:v). Bands were excised and radiolabeled steroids analyzed by scintillation counting using IRGASAFE Plus cocktail (Zinsser Analytic, Frankfurt am Main, Germany) and a β-counter (Packard, Connecticut, USA). Relative conversion was calculated based on total counts.

### CYP17A1 17,20-lyase activity assay

Microsomes, 250 µg/mL, expressing CYP17A1, POR and cytochrome b5 were incubated in reaction buffer for 10 min with 1 mM NADPH and the azole antifungals. The reaction was started by adding 500 nM 17α-hydroxypregnenolone in a total reaction volume of 50 µL. After 1 h of incubation at 37 °C, the reaction was stopped adding 50 µL methanol. Samples were evaporated to dryness, reconstituted in 6 µL methanol, diluted 1:25 in water and the DHEA concentration was quantified using an ELISA kit (DEH3344, Demeditec, Kiel, Germany). Absorbance was measured using a BioTek Citation5 imaging reader.

### Preparation of HEK-293 cell lysates and 3β-HSD2 activity assay

HEK-293 cells (2,000,000) were seeded 24 h prior to transfection in a 10 cm dish. For transfection, 8 µg plasmid DNA were mixed with 500 µL BEST buffer (275 mM NaCl, 1.5 mM Na_2_HPO_4_, 50 mM N,N–Bis(2–hydroxyethyl)-2-aminoethanesulfonic acid, pH 7.0), 62.5 µL 2 M CaCl_2_ and water in a final volume of 1 mL and added to the cells dropwise. After 48 h, cells were washed with PBS before they were collected in 2 mL ice cold PBS. The cell suspension was centrifuged for 4 min at 4 °C at 16,000 × g. The supernatant was aspirated, pellets were shock-frozen in liquid nitrogen and stored at −80 °C. Prior to the enzyme activity assay, a pellet was resuspended in 200 µL ice-cold TS2 buffer (100 mM NaCl, 1 mM EGTA, 1 mM EDTA, 1 mM MgCl_2_, 250 mM sucrose, 20 mM Tris HCl, pH 7.4) and lysed with 10 sonication pulses using an ultrasonic probe. A lysate dilution yielding 10 ng/mL of progesterone formation from 500 nM pregnenolone in 1 h was determined. This dilution was used for all cell pellet aliquots of the same batch. For the enzyme activity assay, the determined cell lysate volume was preincubated with 10 µM of the compounds of interest in TS2 buffer for 8 min. The reaction was started by adding 1 mM NAD^+^ and 500 nM pregnenolone in a final volume of 50 µL. After 1 h of incubation at 37 °C under agitation, the enzyme was heat-inactivated at 95 °C for 1 min. Progesterone concentration was quantified using an ELISA kit (DE1561, Demeditec, Kiel, Germany) on a BioTek Citation5 imaging reader.

## Results

### Forskolin-stimulated H295R cells and effects of prochloraz on the steroid profile

H295R cells were incubated with forskolin to stimulate steroidogenesis and various concentrations of azole antifungals, followed by quantification of nine adrenal steroids in the cell culture supernatant using a targeted UHPLC-MS/MS method. Analysis of the complete medium control (t = 0) revealed that all steroids, except androstenedione, could be detected (see absolute values shown at the bottom of Table 2). The DMSO control represents the steroid production after 48 h under basal conditions. All steroids could be quantified and their levels increased, as expected, following stimulation by forskolin.

**Table 2:** Effects of azole antifungals on the steroid profile of forskolin-stimulated H295R cells.

	

H295R cells were incubated with 10 μM forskolin (stimulated cell control, FC) and azole fungicides at the concentrations indicated for 48 h. The complete medium at the start of the experiment was included as control (MC). Steroids were quantified by UHPLC-MS/MS. Experiments were performed three times independently in duplicates. Absolute concentrations are shown for the complete medium (MC), forskolin control (FC) and unstimulated cells (DMSO control) at the bottom of the Table. Values for the azole antifungals were normalized to the forskolin control. (Data represent mean ± SD, depicted in green in case of a significant downregulation or red when significantly upregulated. Statistical analysis was performed by one-way ANOVA followed by Dunnett’s test and Bonferroni correction. Differences with *p* < 0.05 were considered significant. n.d., not detected.

As suggested by the OECD guideline (Blystone et al., 2007, Hecker et al., 2011, OECD, 2011) prochloraz was included as positive control for the inhibition of steroidogenesis. In line with earlier studies, treatment of the cells with prochloraz resulted in a significant accumulation of progesterone and, less pronounced, of 17α-hydroxyprogesterone and 11-DOC (Ohlsson et al., 2009, Patt et al., 2020, Jäger et al., 2023). In contrast, the levels of glucocorticoids and adrenal androgens downstream of CYP17A1 and CYP21A2 were significantly reduced, which was supported by the inhibitory effects seen in the enzyme activity assays (Table 3). In addition, corticosterone and aldosterone were also reduced, in line with earlier evidence for an inhibition of CYP11B1 and CYP11B2 by prochloraz (Jäger et al., 2023).

**Table 3:** Inhibition of CYP17A1 and CYP21A2 by triazole antifungals.

IC_50_ [µM]	CYP17A1 17α-hydroxylase		CYP17A1 17,20-lyase		CYP21A2	
	mean	SD	mean	SD	mean	SD
prochloraz	0.09	0.03	0.04	0.02	0.21	0.06
posaconazole	1.12	0.33	0.28	0.19	> 10	
itraconazole	> 10		5.76	0.16	> 10	
OH-itraconazole	7.16	0.81	2.48	0.27	> 10	
isavuconazole	> 10		5.71	0.81	8.14	2.64
voriconazole	> 10		> 10		> 10	
fluconazole	> 10		> 10		> 10	

Cell-free enzyme activity assays were performed and inhibition of CYP17A1 17α-hydroxylase and 17,20-lyase as well as CYP21A2 was analyzed. IC_50_ values are given in µM. Data were from at least three independent experiments and represent mean ± SD.

### Application of the H295R model to detect triazole antifungals with a risk of pseudohyperaldosteronism

**Fig. 3:**
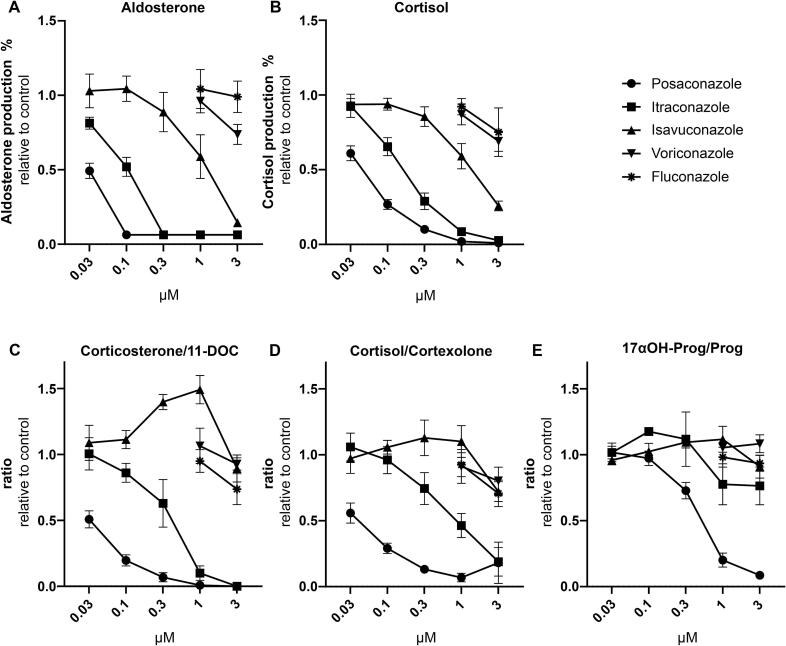
Concentration-dependent effects of selected azole antifungals on aldosterone and cortisol production and on CYP11B1 and CYP17A1 17α-hydroxylase product/substrate ratios. Forskolin-stimulated H295R cells were incubated for 48 h with different concentrations of the selected azole fungicides. Steroid concentrations in the cell culture supernatant were quantified using UHPLC-MS/MS. Aldosterone and cortisol biosynthesis is expressed relative to the forskolin control (A and B, respectively). For samples where the aldosterone concentration could not be quantified, the lower limit of quantification value was included (which was 0,0636 ng/mL, Table 1). The ratios corticosterone/11-DOC (C) and cortisol/cortexolone (D) illustrate the apparent CYP11B1 activity, whereas 17α-hydroxyprogesterone/progesterone (17αOH-Prog/Prog, E) reflects the apparent CYP17A1 17α-hydroxylase activity. Experiments were performed three times independently and represent mean ± SD.

Next, the five systemic azole antifungals were investigated for their potential to inhibit CYP11B enzymes and corticosteroid production. Since adrenocortical toxicity is not included in the routine pre-clinical drug safety testing, the H295R model was applied to retrospectively assess whether the risk of pseudohyperaldosteronism by posaconazole and itraconazole could have been predicted. The biggest changes in the steroid profile were detected for posaconazole, *i.e.*, about 40% and 50% lower levels of the CYP11B1 products cortisol and corticosterone, and the CYP11B2 product aldosterone, respectively, already at the lowest tested concentration of 30 nM (Table 2). This inhibition was concentration-dependent with complete inhibition at 300 nM and higher concentrations (Fig. 3A and B). Itraconazole also inhibited the production of corticosterone and aldosterone and, to a lesser extent, of cortisol in a concentration-dependent manner (Table 2, Fig. 3A and B). Product to substrate ratios can provide useful information on enzyme activities of single enzymes in more complex systems. Both the corticosterone/11-DOC and cortisol/cortexolone ratios, informative of CYP11B1 activity, decreased with increasing concentrations of posaconazole, and less potently itraconazole (Fig. 3C and D). The aldosterone/11-DOC ratio, indicative of CYP11B2 activity, was not calculated because most of the aldosterone levels were below the lower limit of quantification (LLOQ). Nevertheless, the results of the H295R steroid patterns successfully predicted the previously reported inhibition of CYP11B1 and CYP11B2 using specific enzyme activity assays, with a more potent inhibitory effect by posaconazole against CYP11B1 compared to itraconazole (Beck et al., 2020a).

Whilst the exposure to isavuconazole led to a concentration-dependent decrease in all measured steroids, including aldosterone, corticosterone and cortisol, the product/substrate ratios cortisol/cortexolone, corticosterone/11-DOC and aldosterone/11-DOC did not indicate an inhibition of CYP11B1 and CYP11B2, respectively (Table 2, Fig. 3). Furthermore, voriconazole and fluconazole, tested only at the highest two concentrations, showed only minor effects on the steroid profile. These results are in line with the earlier study using enzyme activity assays (Beck et al., 2020a).

**Fig. 4:**
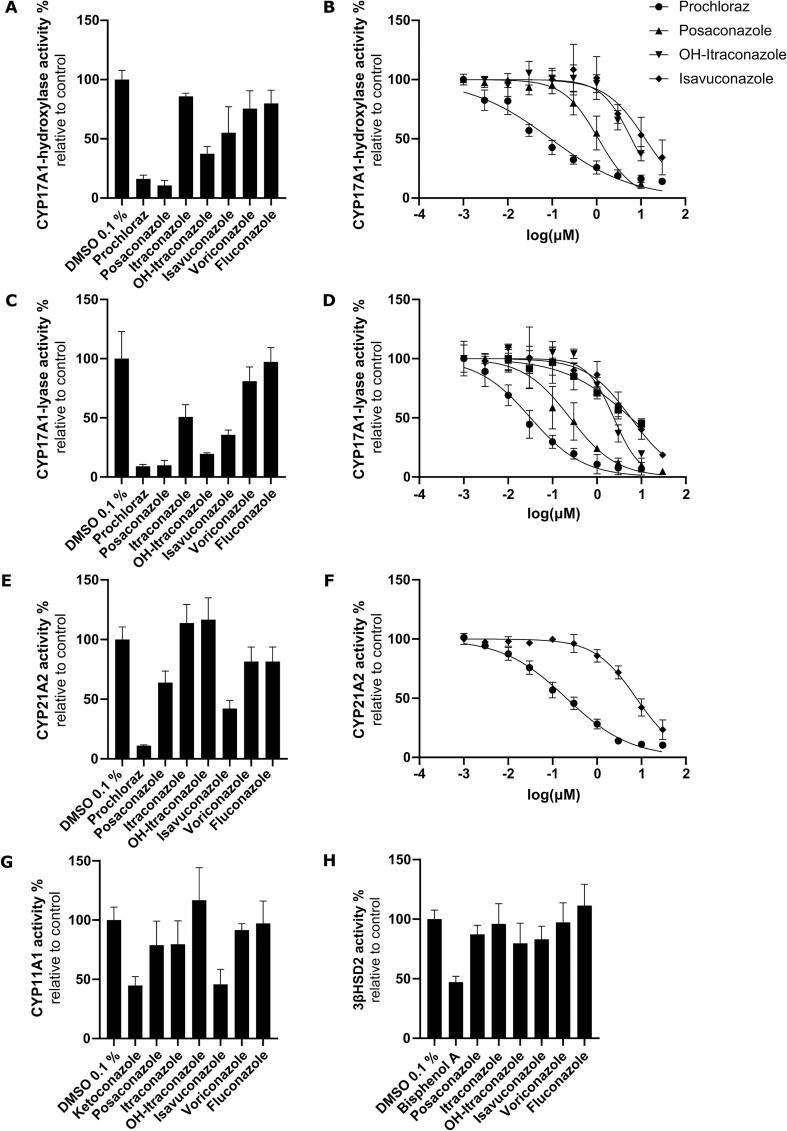
Inhibition of CYP17A1, CYP21A2, CYP11A1 and 3β-HSD2 by selected azole antifungals. The activities of CYP17A1 17αhydroxylase and 17,20-lyase, CYP21A2, CYP11A1, and 3β-HSD2 were assessed by measuring the conversion of progesterone to 17α-hydroxyprogesterone (A and B), 17α-hydroxypregnenolone to DHEA (C and D), progesterone to 11-DOC (E and F), 20α-hydroxycholesterol to pregnenolone (G) and pregnenolone to progesterone (H), respectively. Azole antifungals were initially tested at 10 µM (A, C, E, G, H). Experiments were performed at least three times independently. Data represent mean ± SD.

Another mechanism leading to pseudohyperaldosteronism involves the inhibition of CYP17A1 17α-hydroxylase and the subsequent decrease in cortisol biosynthesis, which then results in a feedback activation of the adrenal steroidogenesis and causes mineralocorticoid excess (Beck et al., 2020b, Beck and Odermatt, 2021). The steroid profiles of H295R cells treated with posaconazole, itraconazole and isavuconazole revealed a concentration-dependent decrease in 17α-hydroxyprogesterone. However, in contrast to posaconazole, where the CYP17A1 substrate progesterone as well as 11-DOC remained unaffected, itraconazole and isavuconazole caused an overall decrease in steroidogenesis (Table 2). Importantly, the product/substrate ratio 17α-hydroxyprogesterone/progesterone indicated 17α-hydroxylase inhibition by posaconazole but not the other systemic azole antifungals (Fig. 3E). This was then confirmed by conducting a 17α-hydroxylase activity assay using microsomes from COS-1 cells expressing recombinant CYP17A1 and POR. An IC_50_ value of 1.12 µM was obtained for posaconazole against 17α-hydroxylase while the other systemic azole antifungals did not inhibit (Fig. 4, Table 3). This represents a moderate inhibition compared to the positive control prochloraz with an IC_50_ value of 94.5 nM.

Thus, the results from the H295R model predict the inhibition of CYP11B1 and CYP11B2 by posaconazole and itraconazole, and revealed an inhibition of CYP17A1 17α-hydroxylase by posaconazole. Furthermore, the use of product/substrate ratios provided important information on the enzymes that were inhibited, which could then be confirmed by the cell-free activity assays.

### Insights beyond pseudohyperaldosteronism

Based on previous studies showing that posaconazole and itraconazole both can cause pseudohyperaldosteronism but that posaconazole more potently inhibits CYP11B1 (Beck et al., 2020a, Beck and Odermatt, 2021), similar steroid patterns were expected after incubating H295R cells to these azole antifungals, yet at different concentrations. However, the steroid profiling of the present study allowed their distinction. While itraconazole as well as isavuconazole lowered the levels of all steroids in a concentration-dependent manner, posaconazole did not affect progesterone and 11-DOC levels (Table 2). Both steroids are synthesized independently of CYP17A1. Because CYP17A1 17α-hydroxylase was moderately inhibited in the cell-free activity assay by posaconazole but only very weakly by hydroxyitraconazole and isavuconazole and not any of the other triazole antifungals tested (Fig. 4A and B, Table 3), the 17,20-lyase activity was assessed in addition (Fig. 4C and D, Table 3). In line with an earlier study (Yates et al., 2017) posaconazole potently inhibited the 17,20-lyase, with a four-fold preference over the 17α-hydroxylase activity. In contrast, hydroxyitraconazole, itraconazole and isavuconazole exhibited 10 and 20 times lower inhibitory potency than posaconazole (Table 3). This was indicated by the strongly reduced levels of DHEA and androstenedione after posaconazole treatment but weaker and much weaker effects by itraconazole and isavuconazole, respectively (Table 2).

Analyses of CYP21A2 and 3β-HSD2 activities in cell-free assays revealed a weak inhibition of CYP21A2 by isavuconazole, with an IC_50_ of 8.1 µM (Table 3, Fig. 4E, F, and H). None of the other systemic triazole antifungals substantially inhibited CYP21A2 or 3β-HSD2 activity. The potent 17,20-lyase and CYP11B1/2 inhibition along with the moderate 17α-hydroxylase inhibition but intact CYP21A2 and 3β-HSD2 activities provide an explanation for the steroid profile of posaconazole. Progesterone and 11-DOC formation is independent of the potently inhibited enzymes. In contrast to prochloraz, with a strong progesterone accumulation and moderate 11-DOC increase due to the potent block of CYP17A1 and CYP21A2, posaconazole only moderately inhibits 17α-hydroxylase and lacks CYP21A2 inhibition. This allows further metabolism of progesterone to 11-DOC as well as some formation of 17α-hydroxyprogesterone and cortexolone.

The H295R steroid profiles following exposure to itraconazole and isavuconazole revealed a general decline in steroidogenesis, suggesting the involvement of another mechanism controlling steroid output. Because CYP11A1 catalyzes the formation of pregnenolone, the precursor of all adrenal steroids, its inhibition would result in a decline of all downstream products. Therefore, a CYP11A1 activity assay using mitochondria prepared from V79 to 4 cells expressing recombinant CYP11A1 and adrenodoxin was established. The imidazole antifungal ketoconazole, reported previously to potently inhibit CYP11A1, was used as positive control (Loose et al., 1983, Mast et al., 2013). However, the cell-free CYP11A1 activity assay used in the present study seemed to exhibit low sensitivity and ketoconazole showed a weak inhibitory effect (Fig. 4G), allowing only preliminary conclusions. Nevertheless, isavuconazole was comparable to ketoconazole in inhibiting CYP11A1 activity. Assuming ketoconazole to be a potent inhibitor of CYP11A1, the comparable inhibitory activity of isavuconazole may provide an explanation for its block of steroid biosynthesis seen in H295R cells (Table 2). Furthermore, posaconazole and itraconazole both showed very weak inhibitory activity against CYP11A1 in the present assay, although posaconazole but not itraconazole was reported earlier to inhibit CYP11A1 (Mast et al., 2013). The assay applied, for unknown reasons, was not sensitive, allowing only qualitative comparison of its inhibition by the azole antifungals. If the inhibitory effects of posaconazole and itraconazole were underestimated in the present study, a moderate CYP11A1 inhibition might contribute to the overall decrease in steroidogenic output following itraconazole treatment and it could prevent an accumulation of progesterone due to CYP17A1 inhibition upon treatment with posaconazole. It needs to be noted that only direct inhibition of steroidogenic enzymes was assessed in this study to decipher the steroid profile obtained with H295R cells. However, effects on cholesterol supply, gene expression or post-translational effects were not covered.

## Discussion

Posaconazole and itraconazole are known to cause pseudohyperaldosteronism with hypokalemia and hypertension by inhibiting CYP11B1 and 11β-HSD2 (reviewed in (Beck and Odermatt, 2021)). Their inhibitory potential towards adrenal steroidogenesis became evident only after market approval and elucidation of the mechanisms underlying the acquired apparent mineralocorticoid excess in affected patients. The present study emphasizes the value of the H295R assay for the identification of compounds interfering with adrenal steroidogenesis. A modified version of the OECD test guideline 456 was applied by using forskolin-stimulated H295R cells to facilitate the identification of inhibitors and quantifying several key adrenal steroids using UHPLC-MS/MS (Hecker et al., 2011, OECD, 2011, Strajhar et al., 2017, Patt et al., 2020). Besides analyzing changes in individual steroid metabolites, product/substrate ratios were determined. This approach enables to detect disturbances on adrenal steroidogenesis, provides initial mechanistic information, and facilitates prioritization of further experimental work. For example, the decreased production of a given steroid metabolite or a decreased product/substrate ratio can provide information on the enzyme responsible for the observed change, either by its direct inhibition or altered gene expression. The underlying mode-of-action can then be investigated by performing activity measurements using recombinant enzyme, determining gene expression and testing for possible changes in post-translational modifications.

In the present study, the known inhibition of CYP11B1 and CYP11B2 by posaconazole and itraconazole (Beck et al., 2020a) could successfully be detected by the decreased concentrations of cortisol, corticosterone and aldosterone. CYP11B1 inhibition by these two azole antifungals was further supported by the evaluation of the corresponding product/substrate ratios corticosterone/11-DOC and cortisol/cortexolone, while there was no such indication for the other three azole antifungals analyzed (Fig. 3). These observations are validated by enzyme activity assays performed earlier (Beck et al., 2020a), showing IC_50_ values (for CYP11B1) of 60 nM, 440 nM and 3 µM for posaconazole, itraconazole, and isavuconazole, respectively. To conclude on patient risk of pseudohyperaldosteronism, inhibition of CYP17A1 17α-hydroxylase and 11β-HSD2 also need to be considered. As reported earlier, posaconazole, itraconazole, and isavuconazole inhibited 11β-HSD2 with IC_50_ of 460 nM, 140 nM and approximately 10 µM, respectively, while voriconazole and fluconazole were inactive (Beck et al., 2017, Beck et al., 2020a). CYP17A1 17α-hydroxylase was moderately inhibited by posaconazole only (IC_50_ 1.12 µM, Table 3). This is likely not of clinical relevance as serum 17α-hydroxyprogesterone levels were normal or slightly elevated in patients treated with posaconazole (Thompson et al., 2017, Barton et al., 2018, Thompson et al., 2019). The slightly increased 17α-hydroxyprogesterone levels are likely a result of the potent 17,20-lyase inhibition (IC_50_ 280 nM, Table 3). In summary, changes in the H295R steroid profiles and enzyme activity data suggest that pseudohyperaldosteronism by posaconazole is mainly caused by CYP11B1/2 and to a lesser extent 11β-HSD2 inhibition, whilst itraconazole predominantly inhibits 11β-HSD2 and to a lesser extent CYP11B1/2. Isavuconazole, voriconazole and fluconazole do not seem to pose a risk of pseudohyperaldosteronism.

The serum trough concentrations for posaconazole, itraconazole, isavuconazole, voriconazole and fluconazole are > 0.7 µg/mL (∼100 nM), >0.5–1 µg/mL (∼100 nM), 4 µg/mL (∼1 µM), 2.5 µg/mL (∼700 nM), and 11 µg/mL (∼3 µM), respectively (Zhang et al., 2018, Märtson et al., 2019, Vena et al., 2020, Risum et al., 2021). For posaconazole an increased risk of pseudohyperaldosteronism was observed at serum concentrations > 2.5 µg/mL (∼350 nM) (Nguyen et al., 2020). The concentrations used in the present cell-based study are in the range of these serum trough concentrations; however, trough concentrations in the adrenals (for CYP11B1 inhibition) or kidneys (for 11β-HSD2 inhibition) likely differ from serum concentrations and involve inter-individual factors, making an extrapolation of our data to the *in vivo* situation difficult.

With respect to posaconazole, the potent inhibition of CYP17A1 17,20-lyase activity provides an explanation for the occurrence of gynecomastia (Thompson et al., 2020). Besides inhibiting the production of adrenal androgens, an inhibition of testicular testosterone formation can be expected, resulting in anti-androgenic effects. No other systemic triazole antifungal showed substantial inhibition of CYP17A1 17,20-lyase activity, suggesting that posaconazole is the only triazole antifungal posing a risk of 17,20-lyase inhibition mediated adverse effects.

The product/substrate ratios partly allowed distinguishing between the mechanisms leading to the overall decrease in steroidogenesis by isavuconazole and itraconazole. While ratios indicated an inhibition of CYP11B1 and CYP11B2 activity by itraconazole already at low treatment concentrations (Beck et al., 2020a), the mechanism leading to the overall inhibition of steroid biosynthesis remains to be determined. In contrast, product/substrate ratios suggest that the mechanism leading to reduced steroid formation by isavuconazole treatment is independent of inhibitory effects on adrenal steroidogenic enzymes. The inclusion of several inhibitor concentrations is important to identify the enzyme that is affected the most. At increasing compound concentrations, inhibition of additional enzymes that possess weaker affinities, contributes to the observed changes in the steroid profile, characterized by broader inhibition of steroid formation at high treatment concentration. Such an effect has been found for itraconazole in the present study and even more pronounced in an earlier investigation with the agricultural azole fungicide epoxiconazole, whose concentration-dependent steroid pattern in H295R cells could clearly be correlated to specific enzyme inhibition (Akram et al., 2019).

Nevertheless, not all of the observed changes in the steroid profiles could be explained mechanistically in the present study. The mechanism underlying the overall inhibition of steroidogenesis by itraconazole and isavuconazole remains unclear. Inhibition of CYP11A1 may provide an explanation. However, in the present study isavuconazole inhibited pregnenolone formation equally efficient as the positive control ketoconazole (Loose et al., 1983, Mast et al., 2013), suggesting similar inhibitory potential of the two compounds towards CYP11A1. Earlier studies found moderate CYP11A1 inhibition by posaconazole (Mast et al., 2013) but no substantial inhibition by itraconazole (Vanden Bossche et al., 1986, Mast et al., 2013), whereas in the present study both compounds showed very weak or no inhibitory activity. Nevertheless, an inhibition of CYP11A1 activity by post-translational modification in the intact cell-system or altered protein stability cannot be excluded. Alternatively, an overall inhibition of steroidogenesis may be caused by an inhibition of cholesterol supply by decreased steroidogenic acute regulatory protein (StAR) activity or impaired mobilization of cholesterol from lipid droplets.

The H295R model and applied approach have limitations. First, even though CYP11B1 and CYP17A1-hydroxylase inhibition can been predicted using the H295R cell model and confirmed by performing cell-free enzyme activity assays, this approach does not cover the HPA axis-dependent feedback mechanism to compensate for the reduced cortisol biosynthesis (Tsigos and Chrousos, 2002). *In vivo*, this feedback stimulation results in the accumulation of cortexolone and 11-DOC, which in excess lead to MR activation and cause hypertension and hypokalemia. Second, inhibition of 11β-HSD2 cannot be assessed by this model due to the low or absent expression. An earlier study failed to detect 11β-HSD2 mRNA levels in these cells (Imamichi et al., 2016) and in the present study UHPLC-MS/MS measurements yielded very low levels of cortisone in the supernatants of cells under basal or forskolin-stimulated conditions (not shown). 11β-HSD2 expression is very low in the adrenals but might be higher in different types of cancer and diseases (Coulter et al., 1999, Mune et al., 2003, Bassett et al., 2005). Third, possible interference with cholesterol synthesis was not assessed. Cholesterol is not present in high concentrations in the here used Nu-serum and *de novo* synthesis by H295R cells might be relevant (Gazdar et al., 1990, Vanparys et al., 2012). Fungal and human CYP51 share about 38% sequence homology (Strömstedt et al., 1996) and weak, unspecific inhibition of the human enzyme was found for all triazole antifungals (Lamb et al., 1999, Trösken et al., 2006, Warrilow et al., 2019, Mercorelli et al., 2020). However, since those pre-clinical studies show that significant inhibition of human CYP51 can be excluded, the observed changes in the steroid pattern by isavuconazole and itraconazole in our study are unlikely caused by a direct inhibition of cholesterol synthesis.

To conclude, the presented approach using forskolin-stimulated H295R cells and steroid profiling along with consideration of enzyme product/substrate ratios proofed useful in identifying compounds inhibiting adrenal corticosteroid biosynthesis and providing initial mechanistic information. The approach successfully indicated CYP11B1 and CYP11B2 inhibition by itraconazole and posaconazole. Furthermore, comparison of the steroid profiles obtained allowed distinguishing the effects of the structurally similar triazoles itraconazole and posaconazole, and indicated inhibition of CYP17A1 17,20-lyase activity by the latter drug. Finally, an overall inhibition of adrenal steroidogenesis by itraconazole and isavuconazole could be identified. Thus, this approach, especially in combination with complementing enzyme activity assays, should prove useful in identifying interferences of compounds with steroidogenesis in early drug development or assessing potential endocrine disrupting chemicals.

## CRediT authorship contribution statement

**Marie-Christin Jäger:** Conceptualization, Investigation, Data curation, Formal analysis, Visualization, Writing – original draft. **Friedrich L. Joos:** Investigation, Formal analysis, Visualization, Writing – review & editing. **Denise V. Winter:** Validation, Writing – review & editing. **Alex Odermatt:** Conceptualization, Validation, Resources, Writing – original draft, Writing – review & editing, Supervision, Funding acquisition, Project administration.

## Declaration of Competing Interest

The authors declare the following financial interests/personal relationships which may be considered as potential competing interests: Alex Odermatt reports financial support was provided by Swiss Centre for Applied Human Toxicology. Alex Odermatt reports a relationship with Swiss National Science Foundation that includes: funding grants.

## Data Availability

Data will be made available on request.
